# Tuning bandgap and surface wettability of NiFe_2_O_4_ driven by phase transition

**DOI:** 10.1038/s41598-018-19319-9

**Published:** 2018-01-22

**Authors:** Sheng-Kai Tong, Po-Wei Chi, Shu-Hsiang Kung, Da-Hua Wei

**Affiliations:** Institute of Manufacturing Technology and Department of Mechanical Engineering, National Taipei University of Technology (TAIPEI TECH), Taipei, 10608 Taiwan

## Abstract

Stress variation induced bandgap tuning and surface wettability switching of spinel nickel ferrite (NiFe_2_O_4_, NFO) films were demonstrated and directly driven by phase transition via a post-annealing process. Firstly, the as-deposited NFO films showed hydrophilic surface with water contact angle (CA) value of 80 ± 1°. After post-annealing with designed temperatures ranged from 400 to 700 °C in air ambience for 1 hour, we observed that the crystal structure was clearly improved from amorphous-like/ nanocrystalline to polycrystalline with increasing post-annealing temperature and this phenomenon is attributed to the improved crystallinity combined with relaxation of internal stress. Moreover, super-hydrophilic surface (CA = 14 ± 1°) was occurred due to the remarkable grain structure transition. The surface wettability could be adjusted from hydrophilicity to super-hydrophilicity by controlling grain morphology of NFO films. Simultaneously, the saturation magnetization (***M***_s_) values of NFO films at room temperature increased up to 273 emu/cm^3^ accompanied with transitions of the phase and grain structure. We also observed an exceptionally tunable bandgap of NFO in the range between 1.78 and 2.72 eV under phase transition driving. Meanwhile, our work demonstrates that direct grain morphology combined with the stress tuning can strongly modulate the optical, surface and magnetic characteristics in multifunctional NFO films.

## Introduction

The heading of wetting characteristics of solid materials surface has been received much attention due to the demonstrating fundamental materials and many promising applications such as green device of environmental cleanup^1,2^ and photonic or optoelectronic devices^3–5^. Moreover, the surface wetting characteristics are usually determined by referring the major data from the measurement of contact angles, indicating the degree of water droplets on the solid surface, this also described the interaction between the solids and liquid. In general, the solid surface with large contact angles (>90°) is corresponded to hydrophobicity, in contract, with small contact angles (<90°) correspond to hydrophilicity. In addition, according to purpose in the 1930s and 1940s by Wenzel, Cassie and Baxter^6,7^, the surface morphology involving micro- or nano-scaled structure was found to play a key role in determining its wetting behavior. It can be understood that the chemical identity of a surface controls its wetting behavior such as carboxylic and hydroxyl functional groups constructing hydrophilic surfaces. On the other hand, the alkyl and perfluoroalkyl groups render surfaces in hydrophobicity. As opposed to easily understood that the surface wetting characteristic is generally governed by the chemical composition and the morphology of solid surface. Meanwhile, the polymetallic oxides with spinel-type structure have attracted much attention due to their chemical and thermal stability are suitable for various applications, such as in biomedical applications as contrast agents in diagnosis^8,9^ and magnetic-guided devices for targeted and pulsed release of active principles^10^. Among of all polymetallic oxides, the nanocrystalline nickel ferrite has unique magnetic properties including superparamagnetism and quantum tunneling of magnetization. Thus, it has been regarded as an excellent candidate in numerous application fields including catalysis^11–13^, sensor technology^14,15^, electromagnetic shielding^16–19^, water treatment^20–23^, and biomedical and biotechnology^24–27^. Fan *et al*. synthesized a carbon nanotube (CNT)/NiFe_2_O_4_−S ternary hybrid material that simultaneously addresses the electron conduction, lithium ion diffusion, and polysulfide dissolution/shuttling problems to boost the performance of the S cathode in Li−S batteries^28^. Li *et al*. prepared the hierarchically porous structures of MFe_2_O_4_ (M = Co, Ni, Cu, Mn) nanofiber with the decreasing mass transport resistances and the both increasing densities and reactivity of exposed electrocatalytic active sites that can be used for various applications in sensing, filtration and adsorption, metal air batteries, energy storage, solar cells, and many other objects^29^. Zeng *et al*. prepared flexible composite spinel-type metal oxide/ reduced graphene oxide (rGO) aerogel materials with much enhanced rate capability, long-term stability and any desired shape that can directly be used as binder-free anodes in lithium ion batteries (LIBs)^30^. Su *et al*. synthesized MFe_2_O_4_ (M = Mg, Ni, Cu) magnetic nanoparticles by using hydrothermal method, which possessed peroxidase-like activity as well as catalase-like activity and used for various fields, such as biotechnology, medical diagnostics and environmental monitoring^31^.

Research works from past till now, most of groups have focused on zero-dimensional (0-D) materials formed by chemical synthesis method, and there is still quite limited research work about the two-dimensional (2-D) nanostructured NiFe_2_O_4_ films directly formed by physical method. On the other hand, the magnetron sputtering technique has attracted much attention due to its advantages for film growth with tunable characteristics, such as easy control for the preferred crystalline orientation and textured growth at relatively low temperature. The above properties are mainly affected by the kinetic energy of the particle bombardment from the plasma during the thin film deposition processes. However, many research works only focused on the physical factors of crystal size, shape and combination with other material characteristics^32–35^. Unfortunately, the work focused on the fundamental surface wetting characteristics of nanocrystalline spinel nickel ferrites films is still lacking. Lüders *et al*. demonstrated the spinel ferrite films growth onto SrTiO_3_ (STO) perovskite as a heterostructure that can be used as the monolithic spin-filter junctions for novel spintronics devices^36^. Kuschel *et al*. showed the Pt/NiFe_2_O_4_ bilayers structure investigated by interface-sensitive x-ray resonant magnetic reflectivity (XRMR), and demonstrated that XRMR is sensitive to the spin polarization at the Pt/FM interface, independent of the Pt film thickness for spintronic applications^37^. On the other hand, as reported by many research groups^38–41^, the nickel ferrites films usually exhibited a lower saturation magnetization value, which plays a key for driving magnetoeletric device. Therefore, in order to solve this issue, a homemade target with composed of NiFe_2_O_4_ powder with 90 wt.% and NiFe powder with 10 wt.% was used. Moreover, to the best of our knowledge, our claimed method for preparing NiFe_2_O_4_ target was in order to enhance the magnetic properties of NiFe_2_O_4_ phase that has never been reported before in published NiFe_2_O_4_ literatures.

In this present article, the spinel NiFe_2_O_4_ films with significantly tunable wettability and bandgap associated with variable internal stress states (see in the Supporting Information, S1) were driven by crystalline phase transition via a directly post-annealing process. We observed that the as-prepared spinel NiFe_2_O_4_ films showed hydrophilic surface with water contact angle (CA) value of 80 ± 1°. After a post-annealing process with designed temperatures ranged from 400 to 700 °C in air ambience for 1 hour, the crystal structure was clearly transformed from amorphous-like/ nanocrystalline to polycrystalline with increasing the post-annealing temperature and this phenomenon can be attributed to the improved crystallinity and grain growth during the crystalline transition process. In addition, the super-hydrophilic surface with a CA value of 14 ± 1° was reached due to the remarkable grain structure transition from sub-nanograin to micrograin type. The surface wettability could be adjusted from hydrophilicity to super-hydrophilicity due to the varied surface free energy caused by controllable grain morphology of NiFe_2_O_4_ nanostructured films combined with stress effect. On the other hand, all samples were displayed a typical magnetic hysteresis loop at room temperature and showed enhanced magnetization compared with that of traditional NiFe_2_O_4_ phase, moreover, the saturation magnetization (*Ms*) values increased with increasing post-annealing temperature. This research demonstrates that a direct crystalline transition combined with stress effect can strongly modulate the optical, surface and magnetic characters in spinel NiFe_2_O_4_ films and provide valuable multifunctional behaviors for potential novel applications.

## Results and Discussion

**Figure 1:**
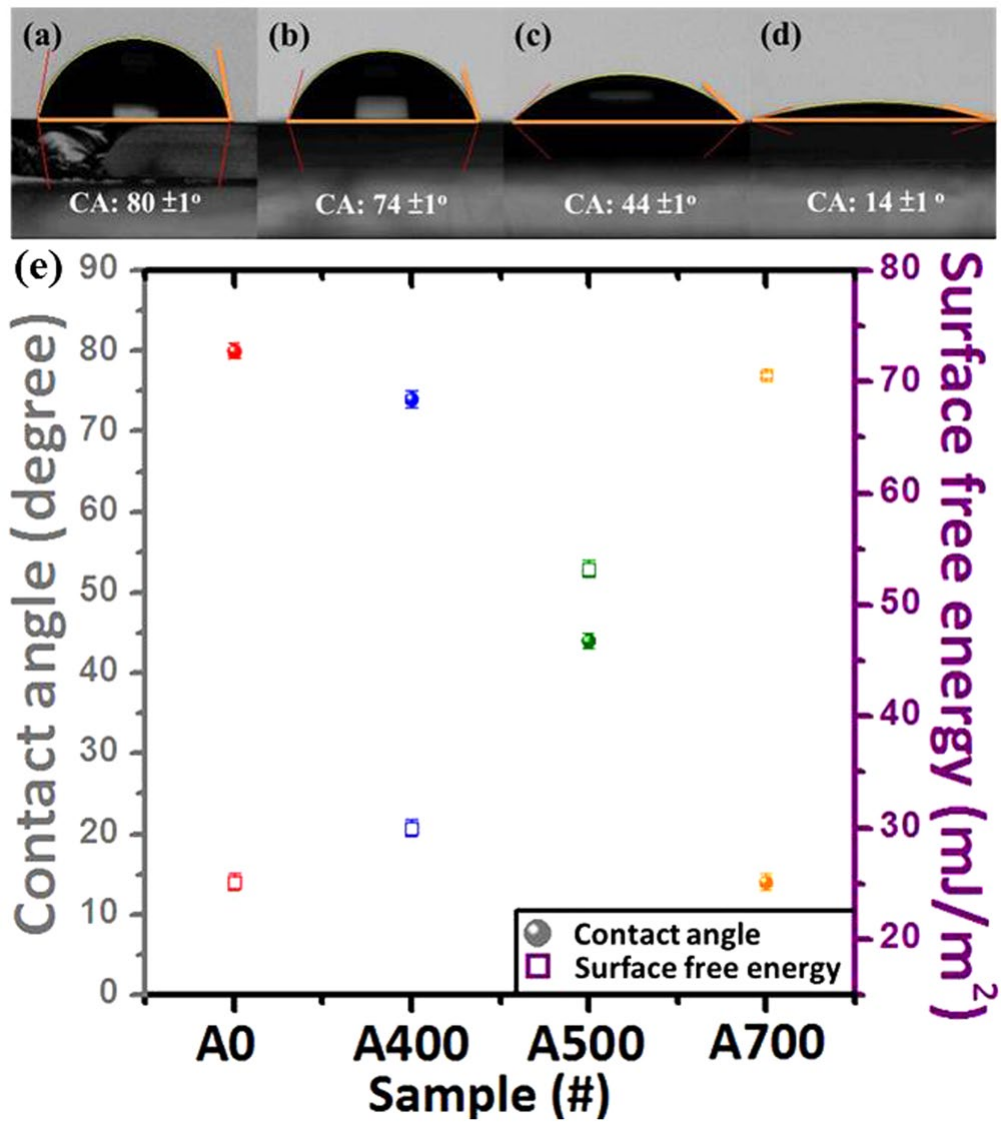
The water contact angle (CA) images for the NiFe_2_O_4_ (NFO) nanostructured films without a post-annealing process and with 400, 500 and 700 °C of post-annealing conditions denoted as (**a**) A0, (**b**) A400, (**c**) A500 and (**d**) A700, respectively. (**e**) The CA and corresponding surface free energy (SFE) values for the above mentioned NFO nanostructured films.

The surface wetting images and corresponding water contact angle for NiFe_2_O_4_ (NFO) nanostructured films without a post-annealing process and with 400, 500 and 700 °C of post-annealing conditions are as shown in Fig. 1(a–d). The values of water contact angles (CAs) are 80 ± 1°, 74 ± 1°, 44 ± 1° and 14 ± 1° for each sample A0, A400, A500 and A700 and the corresponding tendency as shown in Fig. 1(e), respectively. It can be clearly seen that the CAs for the samples A400, A500 and A700 dramatically decrease by 8%, 45% and 83% comparing to that of the as-deposited sample A0, respectively. The above results indicate that the NFO films after a post-annealing process become much more hydrophilic, and the surface wettability of NFO films can be controlled through a simple post-annealing process. Figure 1(e) also shows the surface free energy (SFE) of the designed NFO nanostructured films without and with a post-annealing process. The surface free energy of each sample was calculated by the Fowkes–Girifalco–Good theory, which was mostly used to define the SFE of interface between tested nonpolar liquids and solid surfaces. Relating to the Fowkes method, the dispersive force or van der Waals force is the interaction at the interface between the droplet and solid surface. The Fowkes equation can be described as below:

1 $${\gamma }_{sl}={\gamma }_{s}+{\gamma }_{l}-2{({\gamma }_{s}^{d}{\gamma }_{l}^{d})}^{0.5}$$

where *γ*_*sl*_, *γ*_*s*_ and *γ*_*l*_ are the SFE corresponding to the solid-liquid interface, a solid surface, a measuring liquid, respectively. Where ${\gamma }_{l}^{d}$ and ${\gamma }_{s}^{d}$ are the dispersive portions of the surface tension for the liquid and solid surface, respectively. For further information, the combination of Eq. (1) with the Young’s equation that enables to calculate the SFE of NFO by using a nonpolar liquid (deionized water, *γ*_*l*_ = 72.8 mJ m^−2^), which implies the ${\gamma }_{s}^{d}$ and ${\gamma }_{l}^{d}$ can be equal to *γ*_*s*_ and *γ*_*l*_, respectively, hence the Girifalco–Good–Fowkes–Young (GGFY) equation could be simplified to the following formula:

2 $${\gamma }_{s}^{d}=0.25\,{\gamma }_{l}(1+\,\cos \,\theta )$$

where θ is the measured contact angle between the solid surface and liquid. In this study, the surface wetting states were not only considered from the measured results of contact angles but also calculated the SFE values by using GGFY equation to evaluate all designed NFO samples as shown in Fig. 1(e). At first, the hydrophilic wetting behavior for the NFO film without a post-annealing process (A0) has a surface energy value of 25.1 mJ/m^2^. Once NFO thin films after a post-annealing process became close to super-hydrophilic gradually, and the corresponding surface energy values were varied from 30.1 mJ/m^2^ (A400), 53.2 mJ/m^2^ (A500) to 70.6 mJ/m^2^ (A700). The above results indicated that a strong relationship between the surface wettability and surface energy. It can be clearly seen that the CA value decreased with increasing surface energy for NFO thin films.

The surface wettability of many nanomaterials is almost driven by the tendency to reduce the total Gibbs free energy of the structure via reducing the surface energy part. The detailed description as claimed by our previous work, the aperture on the film surface could induce the air pocket forming and it will let the water drop and easily stand on the surface of as-deposited oxide films^42^. The aperture size and the roughness effect of NFO films without and with a post-annealing process will be investigated and discussed below. Therefore, the grain microstructure plays an important role in surface wettability for solid nanomaterials. It can be understood that after a post-annealing process, the grains of NFO could get more energy to grow up and then the volume of aperture may decrease because when the grains grow up that indicate the amount of the grain boundaries reduced. In other words, it means the number of trap will also decrease then the air pocket will lose, resulting in the CAs tending to much more hydrophilic than without post-annealing one. In order to confirm the micro-structural transition of the grain size and grain boundaries, all the NFO films were observed and checked by FE-SEM. Our present results indicate that the spinel NFO film with tunable surface energy have been successfully demonstrated and controlled by a simple post-annealing process.

**Figure 2:**
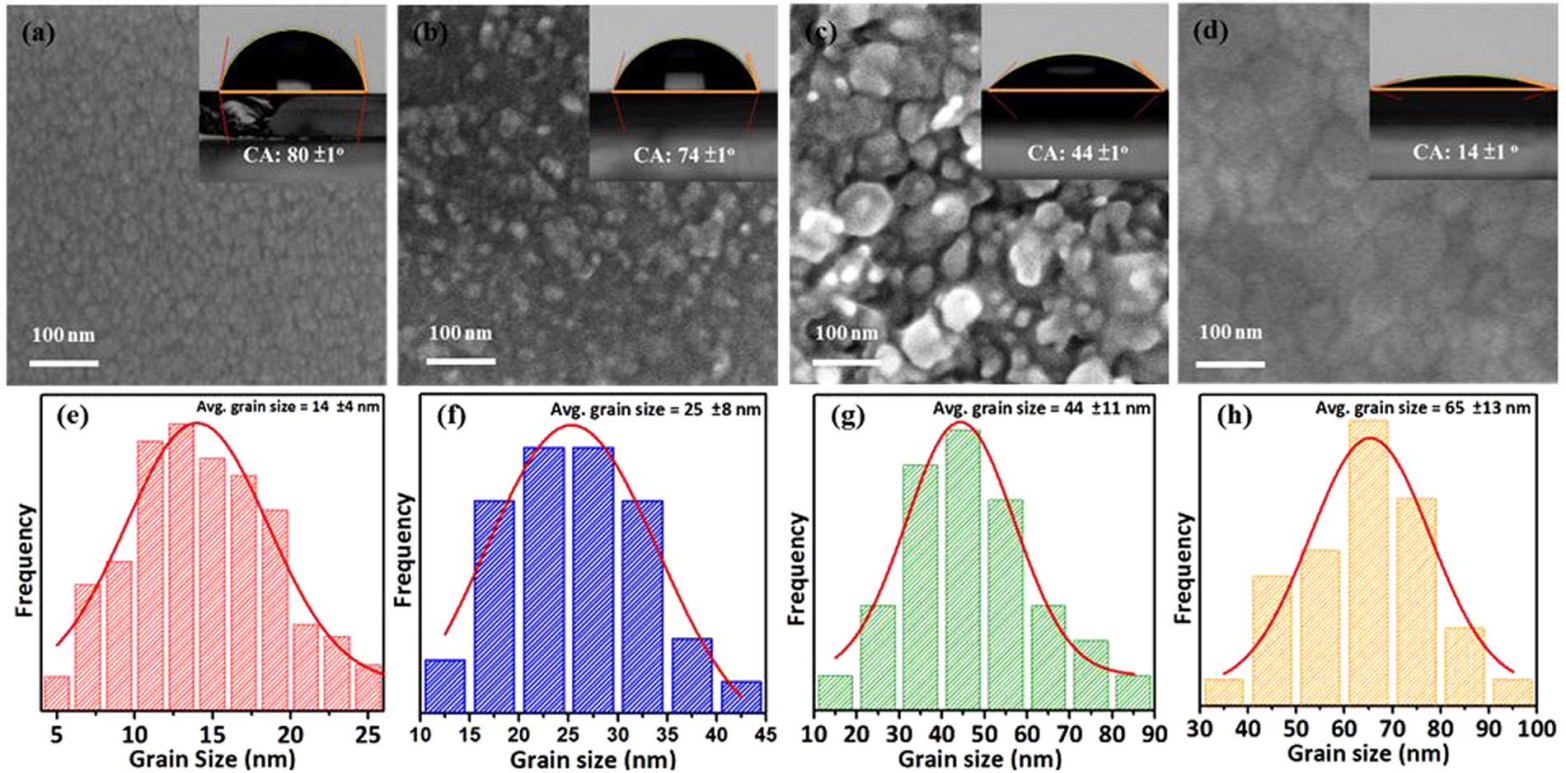
Top view FE-SEM images for the samples (**a**) A0, (**b**) A400, (**c**) A500 and (**d**) A700 NFO nanostructured films, respectively. (**e**–**h**) Show the grain size histograms for evaluating average size and its distribution of the samples A0, A400, A500 and A700, respectively. The insets of (**a**–**d**) are the referred CA images for the samples A0, A400, A500 and A700, respectively.

The top view SEM images for the NFO nanostructured films directly deposited onto glass substrates at room temperature (RT) and with a post-annealing process ranged from 400 to 700 °C as shown in Fig. 2(a–d). The corresponding four different states of grain structures were denoted as sub-nanograin, nanograin, sub-micrograin and micrograin types as shown in Fig. 2(a–d), respectively. The different grain structure was caused by only treating with a varied post-annealing temperature. The as-deposited NFO thin film shows the sub-nanograin structure as shown in Fig. 2(a). On the other hand, the nanograin structure can be observed after a post-annealing temperature of 400 °C as shown in Fig. 2(b). When the post-annealing temperatures were set at 500 and 700 °C, the sub-micrograin and micrograin structures of NFO formed as shown in Fig. 2(c) and (d), respectively. Figure 2(e–h) are the corresponding grain size histograms for evaluating average size and its distribution of the NFO nanostructured films without and with various post-annealing temperatures ranged from 400 to 700 °C. The grains size in fraction of sample A0 ranged from 10 to 18 nm, with an average size of 14 ± 4 nm as shown in the Fig. 2(e), which was denoted as sub-nanograin type. The grains size in fraction of sample A400 ranged from 17 to 33 nm, with an average size of 25 ± 8 nm as shown in the Fig. 2(f), which was denoted as nanograin type. The grains size in fraction of sample A500 ranged from 33 to 55 nm, with an average size of 44 ± 11 nm as shown in the Fig. 2(g), which was denoted as sub-micrograin type. The grains size in fraction of sample A700 ranged from 52 to 78 nm, with an average size of 65 ± 13 nm as shown in the Fig. 2(h), which was denoted as micrograin type. The above results can be attributed to provide the activation energy for leading to the grain transformation and the microstructure coarsening of the NFO thin films, which can be controlled through manipulating annealing temperature^43,44^. It can be observed that with increasing post-annealing temperature, a strong tendency for all grains with multiple domain coalesce and connect to each other to conjoin into a big grain as shown in Fig. 2. On the other hand, it is known that apertures can trap air inside and present onto the surface of NFO thin films. The surface wetting behavior of all samples exhibited with different degree of hydrophilic characteristics. In order to confirm the process of air-pocket forming or losing, the direct evidence to explain the surface wetting behavior of NFO thin films can be checked by AFM images.

**Figure 3:**
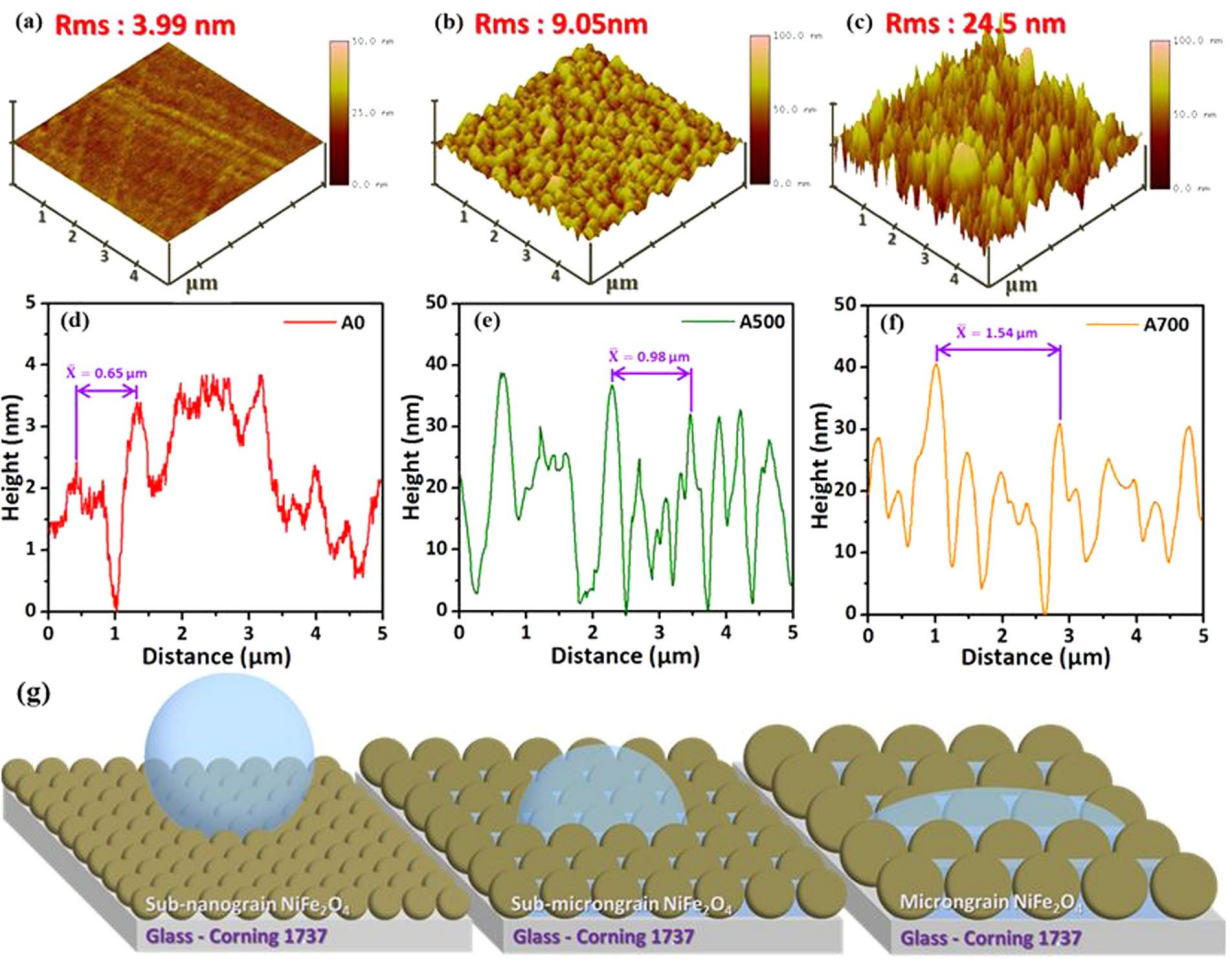
(**a**–**c**) show atomic force microscope (AFM) 3-D images with scanning size of all domain images fixed at 5 × 5 μm^2^. (**d**–**f**) are line profiles illustrating the NFO nanostructured films with different types of grain structure for the samples A0, A500 and A700 NFO films, respectively. (**g**) is the corresponding schematic diagrams of surface wetting behavior with different types for NFO nanostructured films.

Three-dimensional (3D) surface micrographs (5 μm × 5 μm) were observed by using AFM in tapping mode. The topological images of as-deposited NFO films and then post-annealing with different temperatures ranged from 400 to 700 °C as shown in Fig. 3(a–c). The surface topography shows the tendency of the average surface roughness values (root-mean-square, RMS) of NFO films increased with increasing the post-annealing temperature. The average RMS values are 3.99, 9.05 and 24.5 nm corresponding to the samples A0, A500 and A700, respectively. From the above AFM results, it also indicated that the apertures were existed in the NFO nanostructured films. In general, these abundant apertures were sufficient to build up the air pockets and directly affect the surface wettability to be hydrophobic characteristic from the typical Cassie–Baxter theory. Moreover, according to the water droplet onto the natural lotus leaves, it shows such a super-hydrophobic characteristic with water contact angle reached 160° due to their unique nanoscaled structures. In contrast, the wettability of all the samples shows hydrophilic and even super-hydrophilic in our work, because these air pockets existed in NFO nanostructured films were much fewer than general case for the “submicron-scaled” apertures. In addition, during the contact angle measurement, the water droplet will penetrate into the submicron-scaled apertures and substituting the air pockets partially then demonstrate the wettability as hydrophilic. Moreover, the size of apertures were taken into the average values of each sample (A0, A500 and A700) from the AFM line-profile analysis, and the measuring distance was from the center of each sample as shown in Fig. 3(d–f). According to the results, the values of average apertures size are 0.65, 0.98 and 1.54 μm, respectively. It indicated that the average aperture size were gradually increased from 0.65 to 1.54 μm due to the effect of grain growth that the amount of grain boundaries was simultaneously decreased and leading to the larger aperture size. The detailed relationship between average size of apertures and roughness for NFO nanostructured films can be evaluated (see in the Supporting Information, S2)

Figure 3(g) showed schematic diagrams to illustrate the different types of the water droplet on various solid surfaces for NFO nanostructured films, which were transformed from sub-nanograin, sub-micrograin to micrograin types. Tunable grain morphology accompanied with the stress variation that induced from hydrophilicity to super-hydrophilicity of spinel NFO nanostructured films (see in the Supporting Information, S1). So the above transformed surface wetting behavior has been clearly demonstrated and directly driven by phase transition via a post-annealing process. The samples A0 and A400 show hydrophilic wetting, which is due to the relative smooth surface and smaller grain size get less apertures that can not provide the trapping of air denoted as sub-nanograin and nanograin types. While increasing post-annealing temperature to 500 °C, the CA value drastically decreased from 80° (A0) to 44° (A500), which is due to large grain size and more apertures provide more the trapping of air that reduces the contact area between water and smooth surface denoted as sub-micrograin type. The lowest contact angle value (14°) of NFO obtained while increasing post-annealing temperature up to 700 °C (A700), which was due to grain transformation from sub-micrograin to micrograin types and caused the CA decreased originated from the much less apertures formed. The hydrophobic or hydrophilic materials are usually prepared by modifying their surface with varied surface energy for tunable surface wetting characteristics. The surface characteristics for other oxide materials have also been claimed in our previous work^45,46^. In addition, this kind of surface nanostructures could not only be used for self-cleaning^47–49^, drug delivery^50–54^ and biological interactions^55^, but also could be used for other potential surface engineering such as oil-water separation^56–58^, self-dustproof surfaces^59^ and anti-fogging^60^.

**Figure 4:**
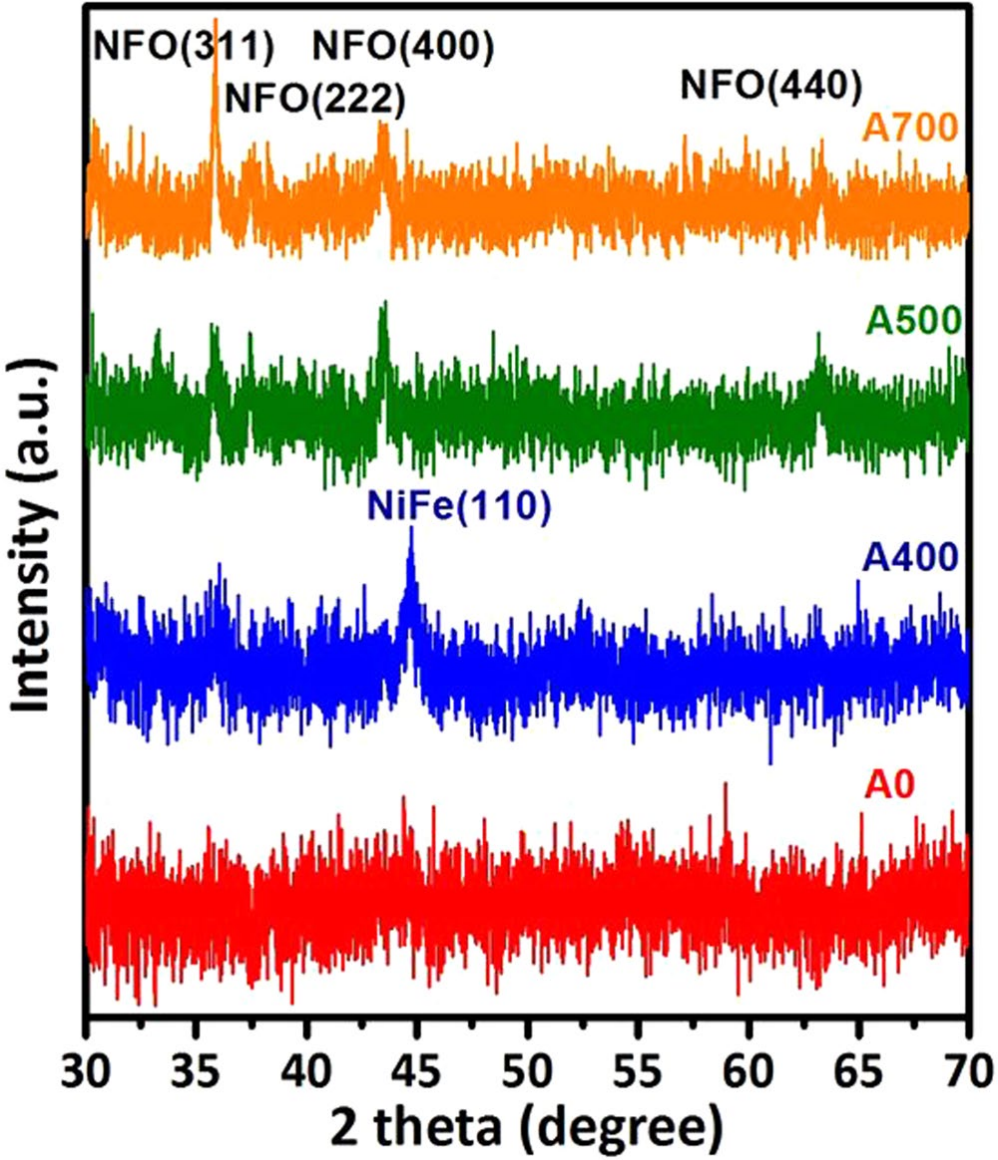
XRD patterns for the samples A0, A400, A500 and A700 NFO nanostructured films, respectively.

The phase formation and crystalline orientation of the NFO nanostructured films without and with post-annealing temperatures ranged from 400 to 700 °C have been identified by using X-ray diffraction as shown in Fig. 4. It can be observed that no significant peaks are existed in the whole diffraction patterns (*θ*-2*θ*) of sample A0, indicating that the crystallization phase state of sample A0 exhibit an amorphous-like/ nanocrystalline structural feature, and it can be proved by SEM image from the Fig. 2(a). For the crystallization phase state of as-deposited NFO, this phenomenon can be attributed to the lack of nucleus activation energy to form the crystalline^43^. The diffraction pattern of the sample A400 shows the peaks of mixed phases with the (311) plane of standard spinel NFO and coexisted phase of NiFe with (110) facet (JCPDS no. 37-0474), which are located at 35.7° and 44.6°_,_ respectively. The NiFe peak is due to our designed NFO target composed of NiFe powder. On the other hand, upon post-annealing to 500 °C, the XRD pattern of sample A500 shows single spinel NFO phase with diffraction peaks located at 2*θ* = 35.7°, 37.3°_,_ 43.4°, and 63°, which are well indexed to the crystal plane of spinel NFO (311), (222), (400), and (440), respectively. From the above results, the phase transition from coexisted phases with NFO and NiFe to single-phase spinel NFO occurred at a critical temperature of around 500 °C, which is due to the filling oxygen atoms combined with NiFe obtained enough driving force to produce the pure NFO grain structure during an optimum post-annealing process. It is noticeable that the intensity of all NFO diffraction peaks significantly increased with the increase of post-annealing temperature up to 700 °C (A700). Moreover, no diffraction peaks of other impurities such as α-Fe_2_O_3_ or NiO are observed from the XRD patterns. On the other hand, it has been clearly shown that the diffraction peaks become sharper and narrower with increasing post-annealing temperature, indicating the enhancement of crystallinity and grain size of the NFO phase, which is consisted with the observation as shown in Fig. 2. According to the above results, indexing that our prepared nanostructured films are belong to the inverse spinel structure with cubic symmetry of the Fd-3m (227) space group and matched well with those of standard NFO (JCPDS no. 89-4927).

**Figure 5:**
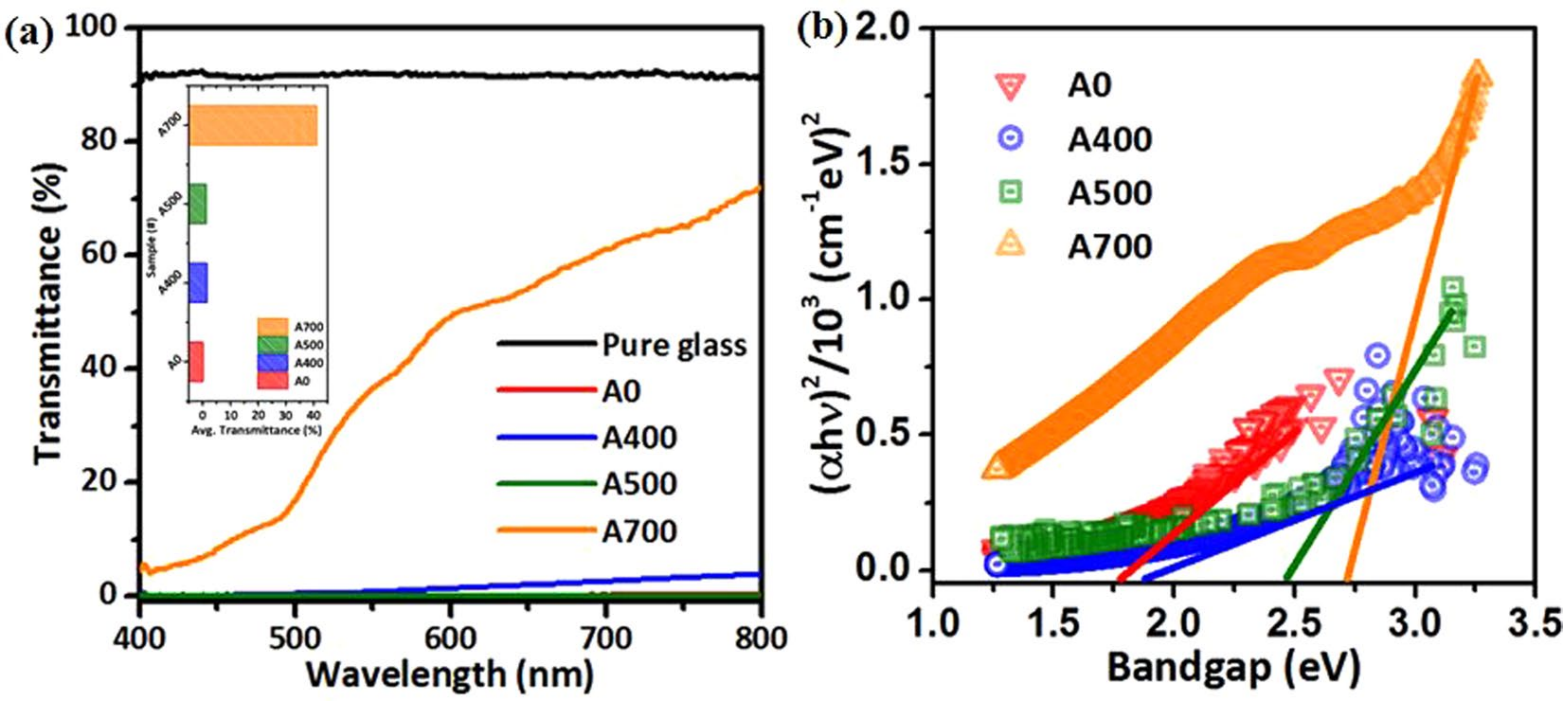
(**a**) Optical transmittance spectra for the pure-bare glass substrate and the samples A0, A400, A500 and A700 NFO nanostructured films, respectively. (**b**) Optical bandgap evaluated from the transmittance spectra using the Tauc’s equation for the samples A0, A400, A500 and A700 NFO nanostructured films, respectively.

The transmittance of the NFO nanostructured films without and with different post-annealing temperatures ranged from 400 to 700 °C as shown in Fig. 5(a). From the transmittance spectra, it can be observed that the samples A0, A400 and A500 represent an opaque characteristic with a visible light averaged transmittance value below approximately 5%. On the other hand, the value of averaged transmittance in visible light increased from 0% to about 42% while rising up the post-annealing temperature up to 700 °C. It can be understood that the higher transmittance was attributed to the higher crystallinity and the fewer defects such as oxygen vacancies, which were filled with oxygen gas from rising post-annealing temperature under ambient air during the post-annealing process. During the post-annealing process, it indicates that the increased crystallinity is associated with the higher annealing temperature due to the much more energy causing the grain growth phenomenon. Besides, the much higher post-annealing temperature also leads to the residual stress relaxation in the NFO nanostructured films and finally induces the grain growth^61,62^ (see in the Supporting Information, S1).

In addition, the determination of optical bandgap of all the samples A0, A400, A500 and A700 for allowed direct transition can be evaluated from the transmittance spectra using the Tauc’s equation described as:

3 $${({\rm{\alpha }}{\rm{h}}{\rm{\nu }})}^{2}={\rm{A}}({\rm{h}}{\rm{\nu }}-{E}_{g})$$

where α is the absorption coefficient, h is Planck’s constant, ν is the frequency of the incident radiation, A is a constant and *E*_g_ is the allowed energy bandgap^63–65^. The Tauc’s plot of (αhν)^2^ versus photo energy (hν) of the NFO nanostructured films without and with different post-annealing temperatures ranged from 400 to 700 °C as shown in Fig. 5(b), and the average bandgap was estimated from the intercept of linear portion of the (αhν)^2^ versus hν plots on hν axis. The bandgap values estimated from the plots for the samples A0, A400, A500 and A700 are 1.78 eV, 1.88 eV, 2.46 eV and 2.72 eV, respectively. The bandgap values were significantly varied from 1.78 to 2.72 eV with a tunable range by different post-annealing temperatures under phase transition driving. This tunable bandgap behavior can be generally interpreted as caused by the phase transition and the partial filled of the oxygen vacancies during post-annealing treatment^66^.

**Figure 6:**
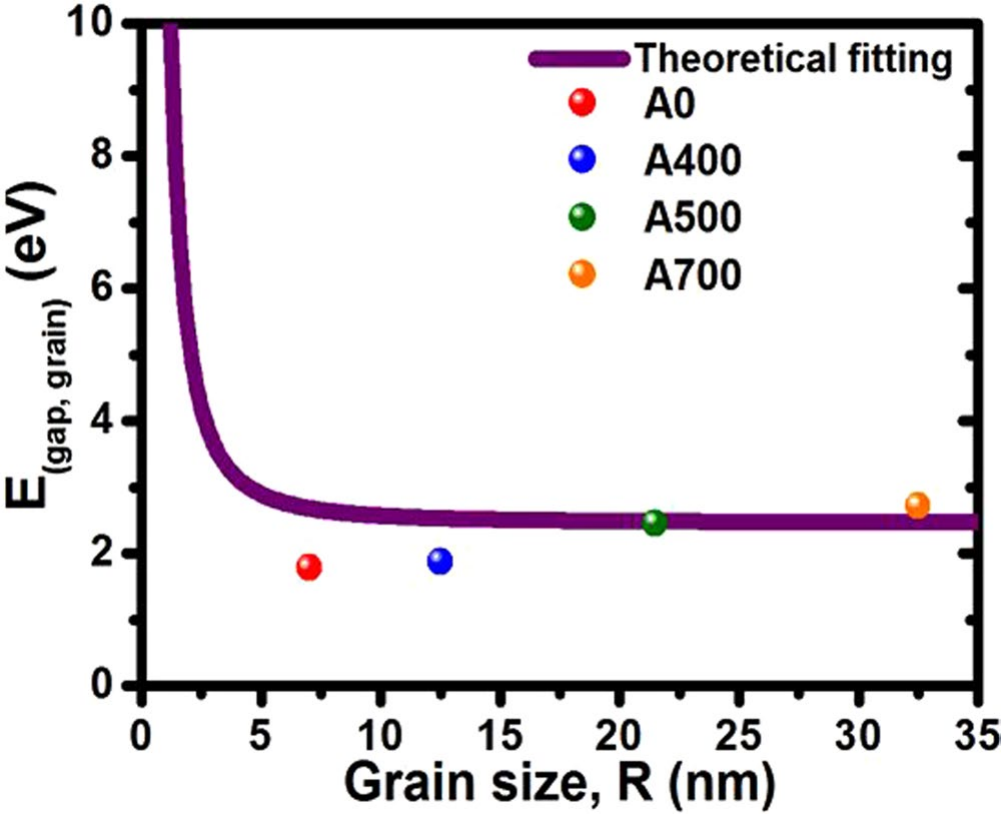
Plot of *E*(gap, grain) obtained from Tauc’s plot as a function of the grain size (grain *radius)* for A0, A400, A500 and A700 NFO nanostructured films.

On the other hand, there are many groups in order to realize the bandgap variation of the oxide nanostructures. Chen *et al*. claimed the blue-shift of ZnO nanorods accompanied by varying in the nanometer size range^67^. Chi *et al*. observed the bandgap shift of ZnO nanostructured films accompanied by changing in size variation combined with internal stress effect^46^. Figure 6 shows a plot of grain band gap *E*_(gap, grain)_ versus the grain size of NFO. The solid curve is theoretical fit of equation (see in the Supporting Information), while the symbols are the values of grain size estimated from SEM images (Fig. 2) and their corresponding bandgap energies evaluated from Tauc’s plot. As shown in Fig. 6, it can be observed that the bandgap energy for the NFO nanostructured films with different grain types evaluated from the Tauc’s plot almost fits the theoretical curve. It is demonstrated that the bandgap energy show theoretical variation with the grain size. In addition, our results also clearly show a bandgap-tunable effect caused by a key factor of internal stress of NFO film via a post-annealing treatment. The above obtained result leads to the conclusion that the variable grain type induced by different internal stress state of the film and the corresponding bandgap of the NFO nanostructured films appears to be related to their grain size on the morphology associated with tunable stress. So based on previous works, the bandgap shifting of the NFO nanostructured films in our present work can be attributed to the quantum-size confinement effect combined with relaxation of internal stress under phase transition driving via a post-annealing treatment. Therefore, the stress/strain modification of material is another possibility for bandgap variation.

**Figure 7:**
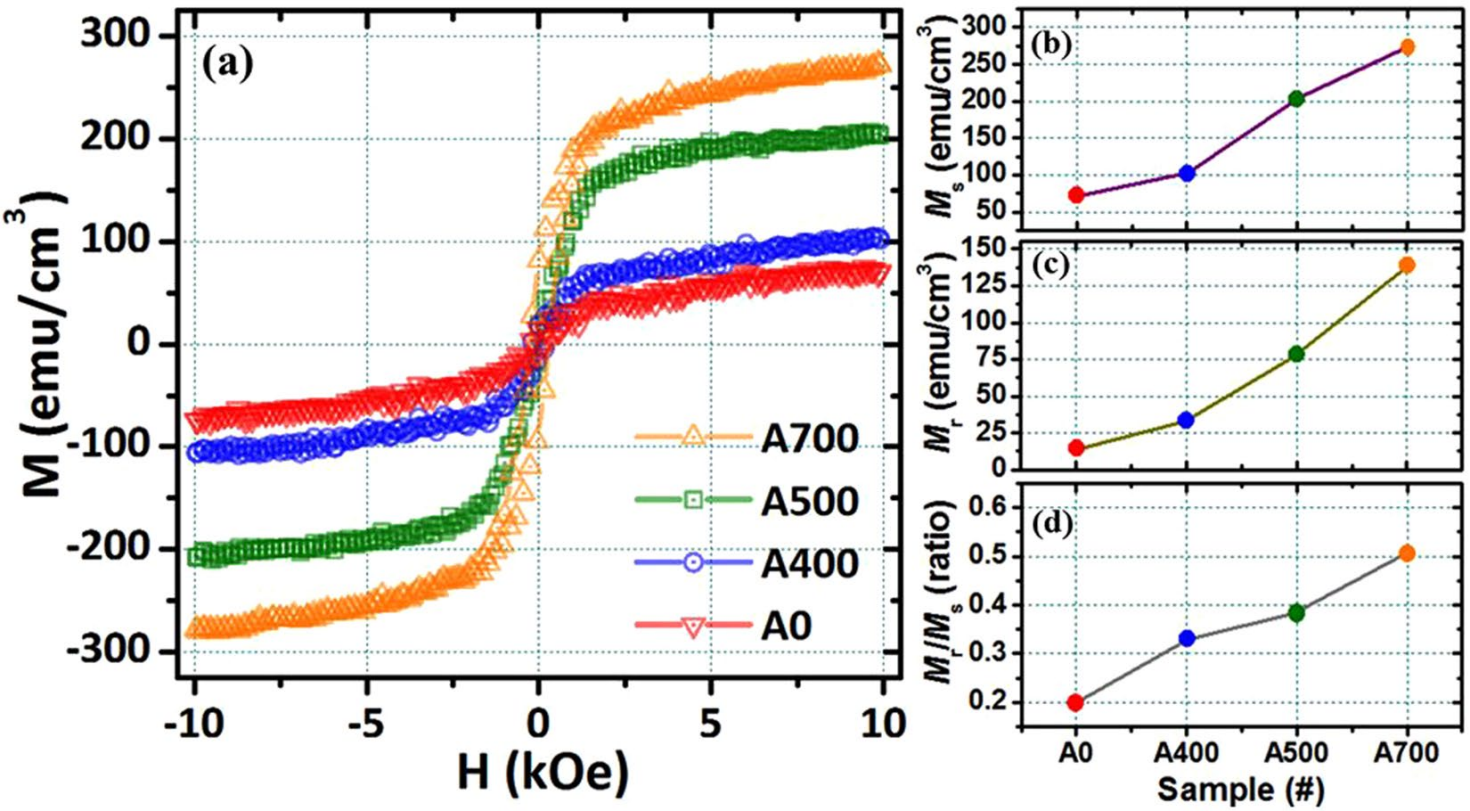
(**a**) The in-plane hysteresis loops measured at room temperature for the samples A0, A400, A500 and A700 NFO nanostructured films, respectively. The corresponding (**b**) saturation magnetization (*M*_*s*_), (**c**) remanent magnetization (*M*_*r*_), and (**d**) remanent squareness ratio (*M*_r_/*M*_*s*_) values for the samples A0, A400, A500 and A700 NFO nanostructured films, respectively.

In order to determine the magnetic behavior of NFO nanostructured films without and with different post-annealing temperatures ranged from 400 to 700 °C, all the magnetic properties have been measured at room temperature with the magnetic field in the plane of the NFO film as shown in Fig. 7. The hysteresis (M-H) loops for samples A0 and A400 are typically superparamagnetic at room temperature as shown in Fig. 7(a). While increasing post-annealing temperature over 500 °C, the NFO films exhibit soft magnetic characteristic under phase transition driving, which is closer towards ferromagnetic nature due to enhanced crystallization quality of pure NFO spinel phase assemblies confirmed by the XRD identification as shown in Fig. 4. The corresponding magnetic data for all NFO nanostructured films including in-plane saturation magnetization (*M*_*s*_), remanent magnetization (*M*_*r*_), and remanent squareness ratio (*M*_r_/*M*_*s*_) values as a function of the NFO films without and with different post-annealing temperatures ranged from 400 to 700 °C are shown in Fig. 7(b),(c) and (d), respectively. The saturation magnetization (*M*_*s*_) values of samples A0, A400, A500 and A700 are increased from 70, 101, 203 to 273 emu/cm^3^ as shown in Fig. 7(b). The remanent magnetization (*M*_*r*_) values of samples A0, A400, A500 and A700 are increased from 14, 33, 78 to 138 emu/cm^3^ as shown in Fig. 7(c). The remanent squareness ratio (*M*_r_/*M*_*s*_) values of samples A0, A400, A500 and A700 are increased from 0.2, 0.32, 0.38 to 0.5 as shown in Fig. 7(d). It can be clearly observed that the *M*_*r*_ and *M*_r_/*M*_*s*_ values of all the samples increased with rising up the post-annealing temperatures ranged from 400 to 700 °C following the increasing tendency of *M*_*s*_. During the crystallization process associated with driving by phase transition, the magnetic property of the NFO films changes from superparamagnetic to ferromagnetic behavior.

Based on the comparison of the *M*_*s*_ value with the previously published literatures^38–41,68^, the saturation magnetization of ideal bulk NFO is 270 emu/cm^3^, our claimed NFO nanostructured films can exhibit excellent magnetic properties with higher saturation magnetization close to theoretical value. The magnetic coercivity (*H*_*c*_) values of NFO nanostructured films without and with post-annealing temperature are increased from 150 Oe (A0) to 292 Oe (A700). Furthermore, it is possible to produce nearly ideal ferromagnetic nanostructured assemblies using the spinel NFO phase. On the other hand, the larger grain size leads the better crystallinity, which induces the better magnetocrystalline anisotropy and finally leads the high saturation magnetization and coercivity, and the similar results also be found by many iron oxide based materials^69,70^. From the above results, it indicates that the better crystallinity can effectively induce the increment of saturation magnetization. The saturation magnetization increment in our study can be attributed to the additive NiFe powder with optimum 10 wt.% in our novelty design target at the beginning.

## Conclusions

In summary, the spinel NiFe_2_O_4_ (NFO) nanostructured films have been successfully fabricated on glass substrates by homemade target using radio-frequency magnetron sputtering system without introducing any oxygen gas during deposition process. The surface wettability of NFO nanostructured films could be tuned from hydrophilic state (80 ± 1° and SFE = 25.1 mJ m^−2^) to super-hydrophilic state (14 ± 1° and SFE = 70.6 mJ m^−2^) through the remarkable transition of grain morphology. The super-hydrophilic surface can be used for many potential applications, such as the oil-water separation, anti-fogging and the self-dustproof surface in order to separate the pollutants from natural environment. In addition, the optical bandgap values were significantly varied in the range between 1.78 and 2.72 eV with technical tunability by different post-annealing temperatures under phase transition driving. The above transformed behaviors combined with relaxation of internal stress have been clearly demonstrated and directly driven by phase transition via a simple post-annealing process. Due to the enhanced crystallization quality associated with phase transition driving by increasing post-annealing temperature, all NFO films displayed a typical magnetic hysteresis loop and the increment of magnetization. In addition, the saturation magnetization (*M*_*s*_) values at room temperature could increased up to 273 emu/cm^3^ that can be attributed to the optimum NiFe powder with 10 wt.% addition in our designed target, and the soft magnetic thin films are needed for developing microinductors and microtransformers. Finally, a simple method has been reported here that the surface wettability and optical bandgap of NFO films can be tuned directly driven by phase transition via a post-annealing process for various potential applications.

## Methods

### Fabrication of NiFe_2_O_4_ nanostructured films

The NiFe_2_O_4_ (NFO) nanostructured films directly deposited onto glass substrates at room temperature were employed by radio-frequency (RF) magnetron sputtering system. In order to enhance the saturation magnetization of the NFO nanostructured films, a homemade target with composed of NiFe_2_O_4_ powder with 90 wt.% and NiFe powder with 10 wt.% was fabricated and used in this study. The target size is with 0.075 m diameter and 0.006 m thickness. All the substrates were set parallel to the target. The Corning-1737 glass substrates (7 × 7 mm^2^) were rinsed in deionized water, ultrasonically cleaned in ethanol and acetone to remove organic contamination then dried in hot air before they load into the sputtering vacuum chamber. The sputtering chamber was pumped down to a base pressure of 5 × 10^−7^ torr. Pure argon was filled into sputtering chamber sequentially with the low working pressure of 10 mtorr. The NFO thin films were deposited with RF power fixed at 50 W with a deposition rate of 5 nm/min. After NFO film deposition, the as-prepared films were annealed under 400, 500 and 700 °C in furnace with fully ambient air for 60 min, then cooling down to the room temperature. Thus, in this article, the as-deposited films without annealing process and with 400, 500 and 700 °C of annealing conditions are denoted as A0, A400, A500 and A700, respectively.

### Characterizations

The crystal structure and crystallinity of the NFO thin films were characterized by X-ray diffraction (XRD) with Cu Kα radiation (λ = 1.54 Å) in the range of 2θ = 30–70°. The surface morphology and microstructure of the NFO nanostructured films were observed by field emission scanning electron microscopy (FE-SEM). The surface topography and roughness values of NFO nanostructured films were further analyzed by the atomic force microscope (AFM). The magnetic properties were measured by a vibrating sample magnetometer (VSM) with an applied field of 10 kOe at room temperature. The wettability of NFO thin films was estimated from the contact angle (CA) of water droplets (10 μl) onto each sample surface. The tolerance of contact angle measurement was affected by image quality with using charge-coupled device (CCD) to capture the image of water droplets and curve fitting function by CA software, which was estimated to be about ±1 degree. On the other hand, the corresponding surface free energy (SFE) could also be calculated and obtained at the same time.

## Electronic supplementary material

Supplementary Information
