# Supplementary material for: Tuning bandgap and surface wettability of NiFe2O4 driven by phase transition
Source: Sci Rep. 2018 Jan 22;8:1338. doi: 10.1038/s41598-018-19319-9 (PMC5778044; doi:10.1038/s41598-018-19319-9)
Supplement: Supplementary file 1 — Supplementary Information [file 41598_2018_19319_MOESM1_ESM.doc]

**Supporting Information**

**Tuning bandgap and surface wettability of NiFe_2_O_4_ driven by phase transition**

Sheng-Kai Tong^†^, Po-Wei Chi^†^, Shu-Hsiang Kung^†^, and Da-Hua Wei^*,†^

^†^ Institute of Manufacturing Technology and Department of Mechanical Engineering, National Taipei University of Technology (TAIPEI TECH), Taipei 10608, Taiwan

**Corresponding Author**

*E-mail: [dhwei@ntut.edu.tw](mailto:dhwei@ntut.edu.tw)

**Internal stress evaluation**

The isotropic biaxial stresses in the films have been determined by comparing the curvatures of the bare glass substrates and substrates coated with a single NiFe_2_O_4_ (NFO) film [1]. In this present study, the internal stress values of NFO films can be given by Stoney’s equation:


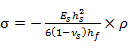
 (1)

where *E_s_* and *ν_s_* are Young’s modulus and Poisson’s ratio of the glass substrate, *h_s_* and *h_f_* are the thicknesses of the glass substrate and NFO film, and *ρ* is the spherical curvature after NFO film deposition. The *h_s_*, *E_s_* and *ν_s_* values of Corning 1737 glass substrate were set as 700 µm, 71 GPa and 0.23, respectively. All NFO films are with a total nominal thickness of 200 nm (*h_f_*) onto glass substrates without any buffer layer confirmed by FE-SEM.

Figure S1 shows the relationship between curvature and the internal stress of the designed NFO samples. It can be clearly observed that nanostructured NFO film is with large tunability of internal stress, which can be either in compressive or tensile stress state. In general, the sign is positive for tensile stress and negative for compressive stress. The NFO films are with varied internal stress in the large range between -5.42 GPa (A0), -3.76 GPa (A400), -1.86 GPa (A500) and 0.47 GPa (A700), respectively.

The thermal energy can lead to relaxation of internal stress and grain boundaries efficiently, indicating an obvious variation in total Gibbs free energy. At the same time, the different microstructure induced by internal stress can be observed as shown in Figs 2(a)-(d), moreover, the thermal energy also assists the phase transition of NFO films as shown in Fig. 4. The tunable surface wettability (hydrophilicity or super-hydrophilicity) was induced by varying stress state directly driven by phase transition via a post-annealing process that can be easily manipulated. Above results indicated that two kinds of compressive or tensile internal stress state can be produced and effectively leaded different wetting behavior on the NFO film surface.


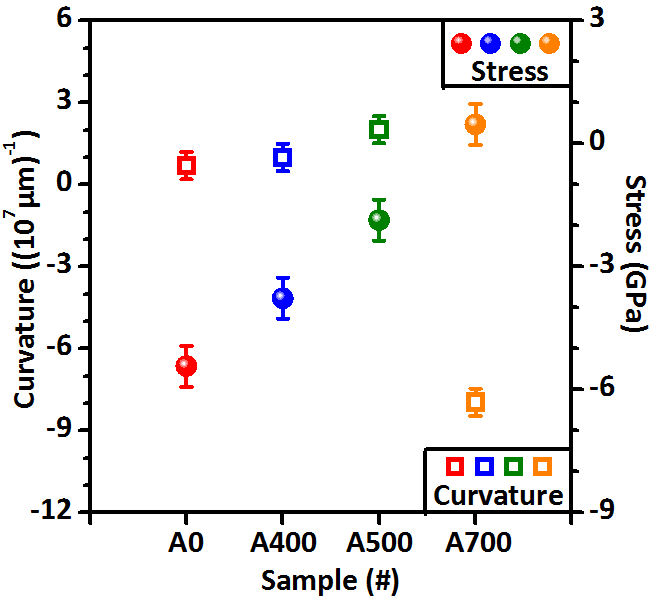


**Figure S1.** The relationship between the curvature (cubic) and internal stress (circle) values for all designed NFO nanostructured films presented as samples A0, A400, A500 and A700, respectively.

**The aperture size and surface roughness (RMS) values for all NFO films**

Figure S2 shows the relationship between the aperture size and average surface roughness (root-mean-square, RMS) values for all NFO films without and with different post-annealing temperatures. The average RMS values are 3.99, 5.85, 9.05, and 24.5 nm corresponding to the samples A0, A400, A500 and A700 NFO films, respectively. The average values of apertures size are 650, 800, 980 and 1540 nm for samples A0, A400, A500 and A700 NFO films, respectively. The surface topography checked by AFM images shows the tendency of the average values of RMS and apertures size of NFO films increased with increasing the post-annealing temperature. Therefore, the surface microstructure should play an important role in wettability for bulk solid materials. The hydrophobic or hydrophilic materials are usually fabricated by modifying their surface with varying their surface energy for forming nano-scaled or micro-scaled structures.

**
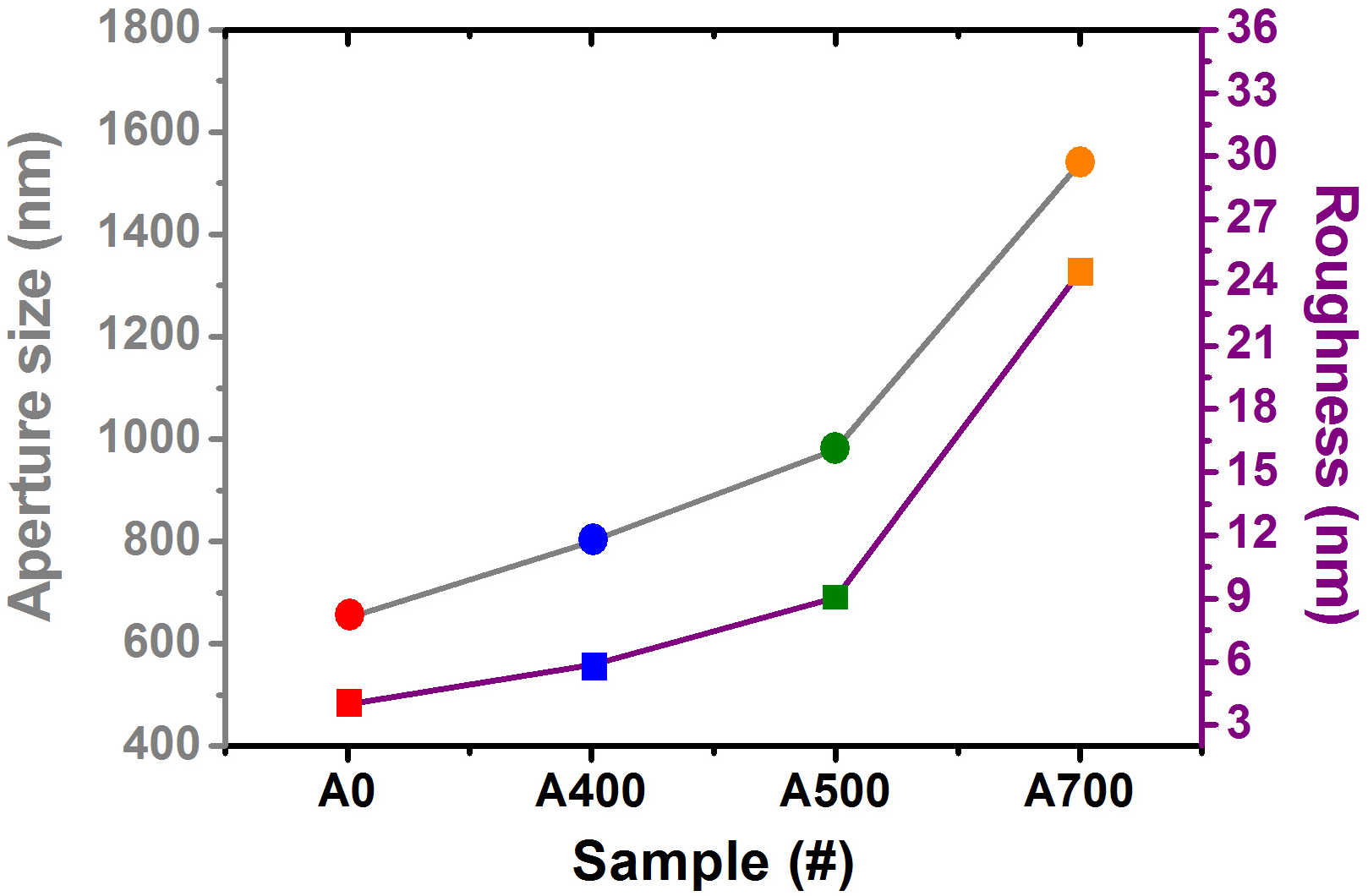
**

**Figure S2.** The relationship between the aperture size and average surface roughness (root-mean-square, RMS) values for all NFO films without and with different post-annealing temperatures ranged from 400 to 700^o^C.

**Theoretical fitting of bandgap energy**

 It is generally known that the band gap energy between conductive band and valence band broadens in nanosystems of semiconductor material, also this kind of phenomenon accompanies with some unique properties in electronics and optoelectronics, as compared with bulk material. Owing to the existence of quantum-size confinement effect, Chen *et al.* reported the blue-shift of ZnO nanostructures accompanied by changing in size variation at the nanometer scale [2]. The band-gap shift and dielectric property enhancement associated with varied internal stress state of ZnO based films have been reported in our previous works [3, 4]. However, the emission by the different grain size will have the similar quantum-size confinement effect as mentioned in this work before and can be described by the following equation:


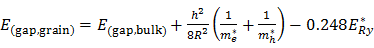
 (2)

where *h* is the Planck’s constant, *R* is the radius of NFO grain,
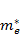
 and
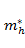
 are the effective masses of electron and hole, as purposed by Beni *et al.* [5] and Tan *et al.* [6], the effective masses of electron and hole are taken
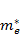
 = 0.24*m*_0_ and
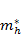
 = 2.31*m*_0_, respectively, *E*_(gap, bulk)_ is the bulk NFO bandgap (2.51 eV [7, 8]), and
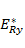
 is the exciton-binding energy in the unstrained structures (28 meV [9, 10]). Based on Eq. 2, the relationship between the grain size (grain radius) and bandgap of NFO nanostructured films can be obtained as shown in Figure 6 (in the manuscript). In addition, our results clearly show a possible bandgap tunable effect caused by a key factor of internal stress of NFO film. Therefore, the stress/strain modification of material is another possibility for bandgap variation.

**Reference**

1. J. Y. Zheng *et al.* *ACS Appl. Mater. Interfaces* **6**, 1351-1355 (2014).
2. C. W. Chen *et al.* *Appl. Phys. Lett.* **88**, 241905 (2006).
3. P. W. Chi *et al. Sci. Rep.* **7**, 43281 (2017).
4. P. W. Chi *et al. J. Mater. Chem. C*, **5**, 1394 (2017).
5. G. Beni *et al. Phys. Rev. B* **18**, 768 (1978).
6. S. T. Tan *et al.* *J. Appl. Phys.* **98**, 013505 (2005).
7. M. Meinert *et al. J. Phys.: Condens. Matter* **26**, 115503 (2014).
8. B. S. Holinsworth *et al.* *Appl. Phys. Lett.* **103**, 082406 (2013).
9. D. Fritsch *et al.* *Appl. Phys. Lett.* **99**, 081916 (2011).
10. M. N. Iliev *et al.* *Phys. Rev. B* **83**, 014108 (2011).
